# Editorial: Advances in Invasive and Non-invasive Brain Stimulation for Dystonia and Other Hyperkinetic Movement Disorders

**DOI:** 10.3389/fneur.2021.741201

**Published:** 2021-08-13

**Authors:** Tommaso Bocci, Maria Knikou, Giacomo Koch, Anna Sadnicka

**Affiliations:** ^1^Azienda Socio-Sanitaria Territoriale (ASST) Santi Paolo and Carlo and Department of Health Sciences, University of Milan, Milan, Italy; ^2^“Aldo Ravelli” Center for Neurotechnology and Experimental Brain Therapeutics, University of Milan, Milan, Italy; ^3^Klab4Recovery Research Laboratory, Department of Physical Therapy, College of Staten Island, The City University of New York, Staten Island, NY, United States; ^4^Ph.D Program in Biology and Collaborative Neuroscience Program, Graduate Center of The City University of New York, New York, NY, United States; ^5^Non Invasive Brain Stimulation Unit, Department of Behavioral and Clinical Neurology, Santa Lucia Foundation Istituto di Ricovero e Cura a Carattere Scientifico (IRCCS), Rome, Italy; ^6^Motor Control and Movement Disorders Group, St George's University of London, London, United Kingdom; ^7^Clinical and Movement Neurosciences, University College London (UCL) Queen Square Institute of Neurology, London, United Kingdom

**Keywords:** dystonia, hyperkinetic movement disorders, deep brain stimulation, transcranial direct current stimulation, repetitive transcranial magnetic stimulation, treatment, Parkinson's disease

Over the last decade, research endeavors have focused on invasive and non-invasive treatments for dystonia and other hyperkinetic movement disorders. However, research outcomes are often limited by their clinical and genetic heterogeneity, the small sample size, and lack of neurophysiological biomarkers compared to Parkinson's Disease (Latorre et al.). In this Research Topic, a range of exciting advances in invasive and non-invasive stimulation for hyperkinetic movement disorders are showcased.

A review by Tisch and Kumar sprovides an overview of globus pallidus deep brain stimulation (DBS) for the monogenic dystonias. The factors that contribute to the variability of outcomes are succinctly summarized and long-term outcomes across the different genetic dystonias (both isolated and combined) are critically appraised. The resultant clinical roadmap of the effect on genes over DBS provides an informative guide to best clinical practice in monogenic dystonias. For example, good outcomes following GPi DBS have been documented in X-linked dystonia Parkinsonism (DYT3), whereas poorer, more variable results have been reported in DYT-THAP1 (DYT6).

Research articles and case histories in this collection also illustrate the expanding number of parameters to consider in the optimization of deep brain surgery. Eleopra et al. retrospectively compares frame-based vs. frameless neurosurgery in 20 patients with dystonia, suggesting that the newer frameless technique is safe, and of comparable efficacy to frame-based surgery and patients' comfort during awake surgery. Neuroanatomically, much research exploring an expanding network implicated in dystonia and tremor has highlighted an important role for the cerebellum. Horisawa et al. detail a rare case in which deep cerebellar stimulation has been recently used after the failure of alternative functional neurosurgeries. With contacts extending from the superior cerebellar peduncle to the dentate nuclei an almost complete resolution of symptoms was achieved with deep cerebellar stimulation highlighting its potential as a treatment for both tremor and dystonia. Fu et al. similarly expands the neuroanatomical network amenable to therapeutic stimulation in Tourette's syndrome. This study examined the effect of bilateral parietal cortex low-frequency repetitive transcranial magnetic with improvements in tic severity and premonitory urges seen both at the end of the 10 days treatment and maintained at 1 month. Prenassi et al. then put the focus on dyskinesia in Parkinson's disease. They probe the observation that amplitude-controlled adaptive DBS can significantly reduce the total amount of electrical energy delivered to the brain and show that dyskinesia was reduced when patients received this adaptive stimulation vs. continuous DBS. The correlation between total electrical energy delivered and the quanta of dyskinesia experienced by patients, therefore, further supports the use of adaptive paradigms to improve patient outcomes.

A common theme is the expansion of the variety and options for stimulation in hyperkinetic movement disorders. A review by Latorre et al. showcases this diversity, discussing features that can be extracted from the oscillatory activity of the central nervous system and how such information can be integrated with brain stimulation. They then shift their focus to the periphery using tremor and Tourette's syndrome to illustrate the utility of peripheral biomarkers and interventions. Finally, the review focuses on current innovations which are changing the landscape of stimulation strategies such as technological advances and the use of machine learning to drive optimization. Correspondingly, Bashir and Wang provides advice on how we can evaluate novel deep learning methods. The authors suggest that all new methods require a comparative justification to other state-of-the-art methods that go beyond the overall accuracy of prediction. They identify computational efficiency and the prediction time for a learning model as core metrics and call for greater detail to be shared within methods so that the broader parameters of models are available for evaluation.

There is much optimism that novel methods of analysis and neural stimulation will transform the management of hyperkinetic movement disorders over the next few decades. This collection of articles provides a range of material, including solid reference resources, novel data, and opinions from leaders in the field, that can be collectively used to optimize therapeutic options for this diverse collection of movement disorders.

## Author Contributions

TB and AS wrote this Editorial. MK and GK revised the final version of the paper. All authors listed have made a substantial, direct and intellectual contribution to the work, and approved it for publication.

## Conflict of Interest

The authors declare that the research was conducted in the absence of any commercial or financial relationships that could be construed as a potential conflict of interest.

## Publisher's Note

All claims expressed in this article are solely those of the authors and do not necessarily represent those of their affiliated organizations, or those of the publisher, the editors and the reviewers. Any product that may be evaluated in this article, or claim that may be made by its manufacturer, is not guaranteed or endorsed by the publisher.

